# Tolcapone induces oxidative stress leading to apoptosis and inhibition of tumor growth in Neuroblastoma

**DOI:** 10.1002/cam4.1065

**Published:** 2017-04-21

**Authors:** Tyler Maser, Maria Rich, David Hayes, Ping Zhao, Abhinav B. Nagulapally, Jeffrey Bond, Giselle Saulnier Sholler

**Affiliations:** ^1^ Pediatric Oncology Translational Research ProgramHelen DeVos Children's HospitalGrand RapidsMichigan; ^2^ College of Human MedicineMichigan State UniversityGrand RapidsMichigan

**Keywords:** Apoptosis, COMT, neuroblastoma, ROS, tolcapone

## Abstract

Catechol‐O‐methyltransferase (COMT) is an enzyme that inactivates dopamine and other catecholamines by O‐methylation. Tolcapone, a drug commonly used in the treatment of Parkinson's disease, is a potent inhibitor of COMT and previous studies indicate that Tolcapone increases the bioavailability of dopamine in cells. In this study, we demonstrate that Tolcapone kills neuroblastoma (NB) cells in preclinical models by inhibition of COMT. Treating four established NB cells lines (SMS‐KCNR, SH‐SY5Y, BE(2)‐C, CHLA‐90) and two primary NB cell lines with Tolcapone for 48 h decreased cell viability in a dose‐dependent manner, with IncuCyte imaging and Western blotting indicating that cell death was due to caspase‐3‐mediated apoptosis. Tolcapone also increased ROS while simultaneously decreasing ATP‐per‐cell in NB cells. Additionally, COMT was inhibited by siRNA in NB cells and showed similar increases in apoptotic markers compared to Tolcapone. In vivo xenograft models displayed inhibition of tumor growth and a significant decrease in time‐to‐event in mice treated with Tolcapone compared to untreated mice. These results indicate that Tolcapone is cytotoxic to neuroblastoma cells and invite further studies into Tolcapone as a promising novel therapy for the treatment of neuroblastoma.

## Introduction

Neuroblastoma (NB) is a tumor of the autonomic nervous system originating from the adrenal medulla and autonomic ganglia in the chest and abdomen 1. After leukemia and brain tumors, NB is the third most frequent malignant tumor in children. Specifically, NB accounts for 15% of all pediatric cancer deaths 2. About 50% of high‐risk patients have relapsed or refractory NB and will succumb to their disease. There are currently no known cures for patients with relapsed or refractory neuroblastoma, with a 5‐year survival of <15% 3.

Approximately 90% of NB patients have elevated levels of catecholamines, specifically dopamine (DA) 4, 5. Multiple studies have indicated that cytosolic DA can undergo oxidation via nonenzymatic mechanisms or by enzymes (such as monoamine oxidase) when DA encounters mitochondria 6. The oxidation of DA results in the production of reactive oxygen species (ROS) 7, 8. In a Parkinson's study evaluating a neuroprotectant, Ouazia et al. (2015) indicated that treatment of a NB cell line (SH‐SY5Y) with DA stimulated the levels of proapoptotic proteins such as cleaved caspase 3 and p53, which causes cell cycle arrest 9. This study additionally showed that when these NB cells were pretreated with antioxidants prior to DA treatment, cleaved caspase‐9 activation was prevented, indicating that apoptosis via accumulation of dopamine is ROS‐dependent in NB cells 9.

Additional studies have shown that increased DA can lead to proteasome inhibition or the regulation of alpha‐synuclein gene via nonoxidative pathways, resulting in mitochondrial dysfunction and cell death. In one study, DA treatment led to the accumulation of alpha‐synuclein and cell death even in the presence of antioxidant N‐acetylcysteine, which supports the hypothesis that depolarization of mitochondria and cell death can occur with an increase in DA via nonoxidative pathways 6, 10.

Catechol‐O‐methyltransferase (COMT) enzyme is found throughout many organs, including brain, liver, kidney, endometrium, and breast tissue 11. Consisting of two isoenzymes in humans, COMT can be membrane‐bound (MB‐COMT) or soluble (S‐COMT) 12. S‐COMT is the predominant form of COMT in the peripheral organs and MB‐COMT is more abundant in the Central Nervous System 13. Physiological substrates of COMT include L‐dopa, catecholamines (DA, norepinephrine, and epinephrine), their hydroxylated metabolites, catecholestrogens, ascorbic acid, and dihydroxyindolic intermediates of melanin 14. Specifically, COMT plays a critical role in the inactivation and metabolism of dopamine and other catechol compounds. The enzyme reduces a catechol molecule in order to prevent genomic damage through DNA adduct formation or via oxygen radicals produced from the redox cycling of catechols 15. In NB, MB‐COMT is localized on the cell surface 16.

Tolcapone is a potent, reversible inhibitor of COMT and the only available COMT inhibitor that is permeable across the blood–brain barrier. Tolcapone is FDA approved in adult patients for the treatment of Parkinson's disease (PD) as an adjunct therapy with levodopa 17, which is a dopamine precursor and is metabolized by COMT. With the concomitant use of Tolcapone, the levodopa absorption time is increased before the drug is metabolized, which allows for increased and sustained motor control for PD patients 18, 19.

**Figure 1:**
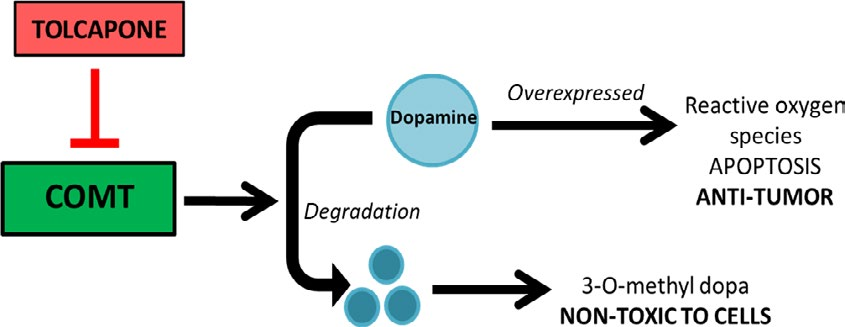
Predicted downstream dopamine pathway of COMT. Inhibition of COMT by Tolcapone results in overexpression of dopamine, leading to apoptosis in NB which characteristically overexpress dopamine.

A previous Parkinson's study using SH‐SY5Y cells indicated that Tolcapone was toxic to human neuroblastoma and caused a significant reduction in ATP synthesis 20. Based on the previous literature and the overexpression of dopamine that is characteristic of NB, we hypothesized that inhibition of COMT by Tolcapone in NB cells would lead to tumor cell death. We predict that by inhibiting COMT, there will be a reduction in dopamine metabolism, resulting in an increased accumulation of dopamine in the NB cells and subsequent release of ROS to induce apoptosis (Fig. 1).

## Materials and Methods

### Inhibitor

The COMT inhibitor Tolcapone (purchased from Sigma‐Aldrich Co. LLC, St. Louis, MO) was dissolved at 100 mmol/L in dimethyl sulfoxide (DMSO). The drug solution was stored in small aliquots at −20°C and diluted with culture media within 2 h of introducing drugs to neuroblastoma cell lines in vitro.

### Cell lines, cell culture, and plating

Single drug tests were conducted on the following NB cell lines: BE(2)‐C (ATCC, Manassas, VA), SMS‐KCNR (graciously donated by Dr. John Maris in 2004, The Children's Hospital of Philadelphia, PA), CHLA‐90 (kindly donated by Patrick Reynolds in 2006, The Children's Hospital of Los Angeles, CA), and SH‐SY5Y (ATCC, Manassas, VA). Cell lines were certified by short tandem repeat (STR) analysis (DNA Diagnostics Center, Fairfield, OH). Drug tests were additionally conducted on two Stage 4 relapsed primary NB patient cells MGT‐015‐08 and MGT9‐102‐08 (Helen DeVos Children's Hospital, Grand Rapids, MI). Cells were cultured in RPMI‐1640 with 10% (vol/vol) certified fetal bovine serum (FBS), 100 U/mL penicillin, and 100 mg/mL streptomycin. Incubation was at 37˚C with 5% CO_2_. All cell culture reagents were purchased from Life Technologies (Grand Island, NY). Cells were grown in 6‐well or 96‐well plates. For all experiments using 96‐well plates, BE(2)‐C, CHLA90, and all preliminary cell lines were plated at 3500 cells/well, and SMS‐KCNR and SH‐SY5Y cells were plated at 5000 cells/well. All plated cells were allowed to adhere overnight and grown to 60–70% confluence within the well prior to drug treatment.

### Cell viability and IC_50_ values

Calcein AM fluorescent assay (Molecular Probes, Eugene, OR) was used to determine cell viability in 96‐well plates. Cells were treated with increasing concentrations of Tolcapone (1.5625–400 μmol/L) in RPMI‐1640 with 10% FBS for 48 h. The concentration of 0.2% DMSO was used as control, consistent with the concentration of DMSO in the highest Tolcapone dose. After 48 h of incubation, the media was aspirated and replaced with phenol‐red free RPMI medium containing 2 μg/mL Calcein AM. After 30 min incubation at 37°C, fluorescence was measured at 480ex/520em with a Synergy HTX multimode plate reader (BioTek Instruments Inc., Winooski, VT). IC_50_ values were calculated with a four‐parameter variable‐slope dose–response curve using GraphPad Prism v.5 software (GraphPad Software Inc., La Jolla, CA).

### BrdU assay

BrdU cell proliferation assay kit (Cell Signaling, Danvers, MA) was used to measure cell proliferation with or without the presence of Tolcapone. Cells were treated with increasing concentrations of Tolcapone (1.5625–400 μmol/L) in RPMI‐1640 with 10% FBS for 48 h. The concentration of 0.2% DMSO was used as control, consistent with the concentration of DMSO in the highest Tolcapone dose. After 48 h of incubation with Tolcapone, BrdU solution was added to the cells and the manufacturer's protocol was followed from there. Absorbance was read at 450 nm with a Synergy HTX multimode plate reader (BioTek Instruments Inc.). Results were normalized to corresponding vehicle wells in order to quantify data. GI_50_ values were calculated with a four‐parameter variable‐slope dose–response curve using GraphPad Prism v.5 software (GraphPad Software Inc.).

### Quantitative RT‐PCR

After 72 h of siRNA transfection, SMS‐KCNR cells were lysed and total RNA was extracted using the RNeasy Mini Kit (Qiagen, Hilden, Germany) per the manufacturer's protocol. Reverse transcription was then conducted using the High Capacity RNA‐to‐cDNA Kit (Applied Biosystems, Foster City, CA) following the manufacturer's instructions. RT‐PCR was performed in triplicate using SsoAdvanced Universal SYBR Green Supermix (Bio‐Rad, Hercules, CA) with primers for both COMT (Bio‐Rad) and the reference β‐actin (Applied Biosystems) gene in a reaction volume of 20 μL. The cDNA was amplified using a CFX96 Real‐Time System (Bio‐Rad), following the recommended cycling parameters (40 cycles of 95°C for 5 sec and 40 cycles of 60°C for 30 sec). The quantification of the changes in COMT gene expression was calculated from the cycle threshold (Ct) value using the Bio‐Rad CFX Manager program.

### IncuCyte imaging assay

IncuCyte imaging of SYTOX Green (Life Technologies, Grand Island, NY) and Kinetic Caspase‐3/7 reagent (Essen Bioscience, Ann Arbor, MI) were used to measure cytotoxicity and apoptosis in NB cells treated with Tolcapone. Cells were plated in black, clear bottom, 96‐well plates. After 24 h incubation, media was aspirated from each well and cells were treated with Tolcapone (25, 50, 75, 100, 200, 400 μmol/L doses) in RPMI‐1640 with 10% FBS and 200 nmol/L SYTOX or 5 μmol/L kinetic caspase‐3 reagent (50% cells treated with SYTOX and 50% treated with caspase‐3 reagent at each Tolcapone dose level). Plates were placed in the IncuCyte ZOOM (Essen Bioscience) at 37°C and imaged over 48 h at 4 h time points for phase and green fluorescence.

### Western blot analysis

Cells were plated in 6‐well plates at a cell density ranging from 100,000 to 500,000 cells/well in RPMI‐1640 with 10% FBS and 2% penicillin‐streptomycin and were allowed to incubate at 37°C for 18‐24 h. The media was then aspirated and established cells were treated with 100, 75, 50, and 25 μmol/L Tolcapone, while primary patient cells were treated with 400, 200, 100, and 50 μmol/L Tolcapone in RPMI with 10% FBS and 2% penicillin‐streptomycin. The cells were allowed to incubate for 48 h at 37°C. Samples were then diluted in RIPA lysis buffer containing protease and phosphatase inhibitors (Cell Signaling) to yield equal protein concentrations within each sample. Cell lysates were then collected and stored at −80°C to confirm complete lysis. Bradford assay (Bio‐Rad) was performed to determine protein concentrations. Equal amount of protein was loaded per lane into a 12% SDS‐polyacrylamide gel in running buffer (Bio‐Rad) and electrophoresed. Gels were then semidry transferred onto a nitrocellulose membrane using the Turbo Transfer system with Turbo Transfer buffer (Bio‐Rad). Primary antibodies include Caspase‐3 (Cell Signaling, 9662S), Cleaved Caspase‐3 (Cell Signaling, 9661L), PARP (Cell Signaling, 5626S), Cleaved PARP (Cell Signaling, 9532S), COMT (Santa Cruz Biotechnology, Inc., Santa Cruz, CA, sc‐25844) and β‐Actin (Cell Signaling, 4967S). Goat antirabbit horseradish peroxidase conjugated secondary antibody was used (Santa Cruz Biotechnology, Inc., sc‐2004). Protein bands were detected by chemiluminescence with the ChemiDoc^™^ MP System (Bio‐Rad). Protein bands of interest were quantified and reported as percent of quantified β‐Actin to account for small differences in loading. Quantification of protein bands was performed using Image Lab^™^ Software, Version 5.2 (Bio‐Rad).

### siRNA transfection

SMS‐KCNR and BE(2)‐C cells were plated in 6‐well plates at a cell density of 300,000 to 500,000 cells/well in RPMI‐1640 with 10% FBS and 2% penicillin‐streptomycin. Cells were allowed to incubate at 37°C for 18‐24 h. Four treatments were administered to the cells: untreated, negative control (vehicle), transfection reagent only, and 50 nmol/L siRNA. A 1:50 dilution of Lipofectamine RNAiMAX (Invitrogen, Waltham, MA) was prepared in Optimem medium (Invitrogen) and incubated at 20˚C for 5 min. The indicated treatment concentration was then prepared in Optimem medium and combined with the 1:50 diluted Lipofectamine solution. The solution was gently mixed and incubated for 20 min at 20°C. While the solution was incubating, media was aspirated off the cells. Cells were then introduced to transfection complex. After the transfection complex was added, 1.5 mL of RPMI‐1640 with 10% FBS and no penicillin‐streptomycin was added to each well, and the cells were returned to the incubator at 37°C. After 24 h of incubation, an additional 1.5 mL of RMPI‐1640 with 10% FBS and 2% penicillin‐streptomycin was added to each well and the cells were then incubated for an additional 48 h. Cells were collected for western blot analysis according to the above protocol.

### ATP‐per‐cell

The Cell Titer GLO luminescent assay (Promega, Madison, WI), which measures total ATP levels, was combined with the CyQuant fluorescent DNA assay (Invitrogen, Grand Island, NY), an indicator of total cellular DNA present, to measure ATP level per cell. Cells were plated in 96‐well black‐walled plates. Cells were treated for 48 h with 50, 75, 100, and 200 μmol/L of Tolcapone in RPMI‐1640 with 10% FBS. The concentration of 0.2% DMSO was used as control, consistent with the concentration of DMSO in 200 μmol/L Tolcapone dose. After 48 h, medium was aspirated and replaced with Cell Titer GLO reagent (lysis buffer mixed with Cell Titer GLO substrate, diluted with PBS 1:1) supplemented with CyQuant stock solution (50 μL dye per 10 mL Cell Titer GLO reagent). Plates were rocked on an orbital shaker for 3 min at 20°C to induce cell lysis, and then were incubated an additional 10 min at 20°C to allow luminescent signal stabilization. Luminescence and fluorescence data were recorded using a Synergy HTX multimode plate reader (BioTek Instruments Inc.). Cell Titer GLO data were divided by CyQuant data and normalized to vehicle wells in order to quantify data.

### ROS assay

DCFDA Cellular ROS detection assay (Abcam, Cambridge, MA) was used to determine the presence of reactive oxygen species for cells treated with Tolcapone in 96‐well plates. Following plating of BE(2)‐C and SMS‐KCNR cells and 24 h of incubation, the media was aspirated, cells were washed with 100 μL of 1× buffer (Abcam) per well, and 100 μL of 20 μmol/L DCFDA (Abcam) dissolved in 1× buffer was added to each well. DCFDA was used to stain the hydroxyl, peroxyl, and other ROS activity within the cells. Cells were incubated at 37°C in the dark for 45 min. DCFDA solution was then removed and replaced with 0, 50, 75, or 100 μmol/L Tolcapone in phenol‐red free RPMI‐1640 with 10% FBS and 2% penicillin‐streptomycin for 1 h and 3 h time points. The concentration of 0.2% DMSO was used as control, consistent with the concentration of DMSO in 100 μmol/L Tolcapone dose. Following incubation of cells at the given time points, dosed media was removed and 50 μL PBS was added to each well. Fluorescence was measured at 480ex/520em with a Synergy HTX multimode plate reader (BioTek Instruments Inc.). ROS measurements were normalized to vehicle wells in order to quantify data.

### In vivo studies

In vivo drug studies were conducted with 4‐week‐old female nude mice (nu/nu) (Charles River Laboratories, Portage, MI). Mice were individually housed in pathogen‐free conditions and cared for in accordance with the Institutional Animal Care and Use Committee (IACUC) standards. All mice received SMS‐KCNR (2 × 10^6^) cells suspended in 100 μL of Matrigel (BD Biosciences, San Jose, CA) via subcutaneous injection into their right flank. Once tumors were palpable (about 10 days postinjection), mice were grouped into two treatment groups with equal average tumor sizes, including nine mice per group: untreated group and 125 mg/kg Tolcapone‐treated group. Tolcapone was supplied in pellets of food formulated with 500 mg Tolcapone/kg of diet (Harlan Sprague Dawley Inc. Indianapolis, Indiana). To achieve the target dose of 125 mg/kg, 5 g of 500 mg tolcapone/kg food was provided to each mouse once every 24 h (125 mg/kg × 0.02 kg mouse weight/500 mg Tolcapone/kg diet = 0.005 kg diet). Mouse weight was recorded weekly and tumor volume measurements were taken tri‐weekly. Once tumor volume reached 2000 mm^2^, tumor volume measurements were taken daily. Mice were followed to survival, and each mouse was euthanized when the tumor volume reached 3000 mm^2^. All tumors were then resected, weighed, and snap froze for future laboratory testing. Mouse survival in treatment group was compared to the mouse survival in the control group. The experiment was replicated three individual times.

### Statistical analysis

All experiments were replicated for statistical strength (*n* = 3). IC_50_ and GI_50_ values were calculated with a four‐parameter variable‐slope dose‐response curve using GraphPad Prism v.5 software (GraphPad Software Inc.). Microsoft Excel was used for fold‐change quantification of protein expression for Western blot analyses. Optic density of each band compared to the normalized actin bands was determined using Image Lab Software (Bio‐Rad). Two‐tailed unequal variance t‐tests were used to determine statistical significance for ATP‐per‐cell reduction and ROS fold‐change with Tolcapone treatment for each independent cell line.

For each of the three in vivo experiments, the primary test of the null hypothesis was a nonparametric comparison of tumor size based on the last observation for which tumor size was evaluable in all mice. Similar results were obtained using parametric tests based on tumor size as well as on time‐to‐threshold tumor size. The results of the three tests were combined using Fisher's method.

## Results

### Tolcapone is cytotoxic to NB cells

**Figure 2:**
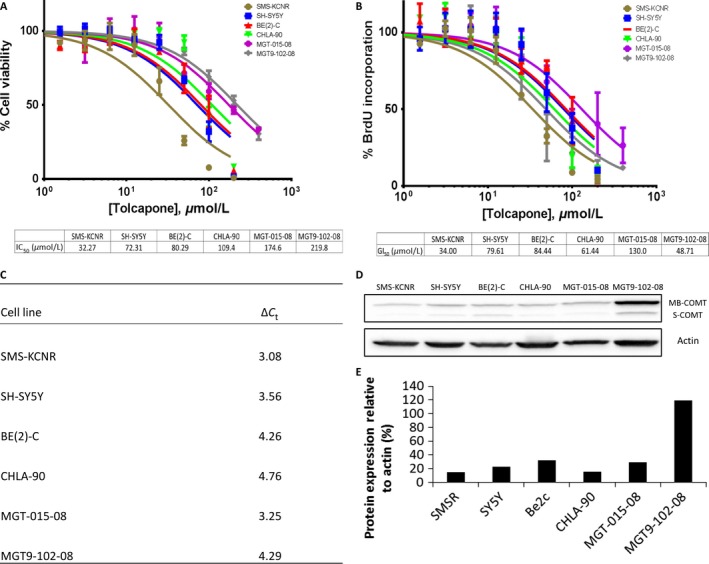
Tolcapone exhibits cytotoxicity as a single agent in four NB cell lines and two primary tumors. (A) IC_50_ values were calculated 48 h postintroduction of Tolcapone drug treatment using Calcein AM fluorescent assays. (B) GI_50_ values were calculated 48 h postinduction of Tolcapone drug treatment using BrdU assays. (C) RT‐qPCR was run on lysates from all cell lines used to determine basal mRNA levels of COMT. ∆CT represents the change in cycle threshold of COMT compared to β‐Actin. (D) Western blot was run on lysates from all cells to determine basal protein levels of MB‐COMT and S‐COMT. (E) Bands were normalized to β‐Actin control and quantified as a percent compared to β‐Actin expression.

Cytotoxicity of Tolcapone as an independent therapeutic treatment for NB cells was determined and quantified using Calcein AM fluorescent assay for multiple NB cell lines (SMS‐KCNR, SH‐SY5Y, BE(2)‐C and CHLA‐90) and NB primary tumors (MGT‐015‐08 and MGT9‐102‐08). Cell viability analysis indicated that Tolcapone is cytotoxic to NB cells with IC_50_ values ranging from 32.27 μmol/L for SMS‐KCNR cells to 219.8 μmol/L for MGT9‐102‐08 primary cells (Fig. 2A).

The cytostatic effect of Tolcapone treatment on NB cells was analyzed using the BrdU assay. Cell proliferation analysis resulted in GI_50_ values ranging from 34.0 μmol/L for SMS‐KCNR cells to 130.0 μmol/L for MGT‐015‐08 primary cells (Fig. 2B). The comparison of data obtained from the cell viability and BrdU assays shows that most cell lines had similar IC_50_ and GI_50_ values when treated with the same doses of Tolcapone (Fig. 2A and B). This suggests that the effect of Tolcapone is primarily cytotoxic to NB cells. Only MGT9‐102‐08 had a significantly lower GI_50_ relative to IC_50_ suggesting an effect on cell proliferation as well as cytotoxicity.

Basal RNA and protein levels of COMT, the enzyme targeted by Tolcapone, were also quantified by RT‐qPCR and Western blotting (Fig. 2C–E). The majority of the COMT appears to be membrane‐bound (MB‐COMT) with minimal soluble (S‐COMT). All primary patient cells and established cell lines analyzed were sensitive to Tolcapone treatment.

**Figure 3:**
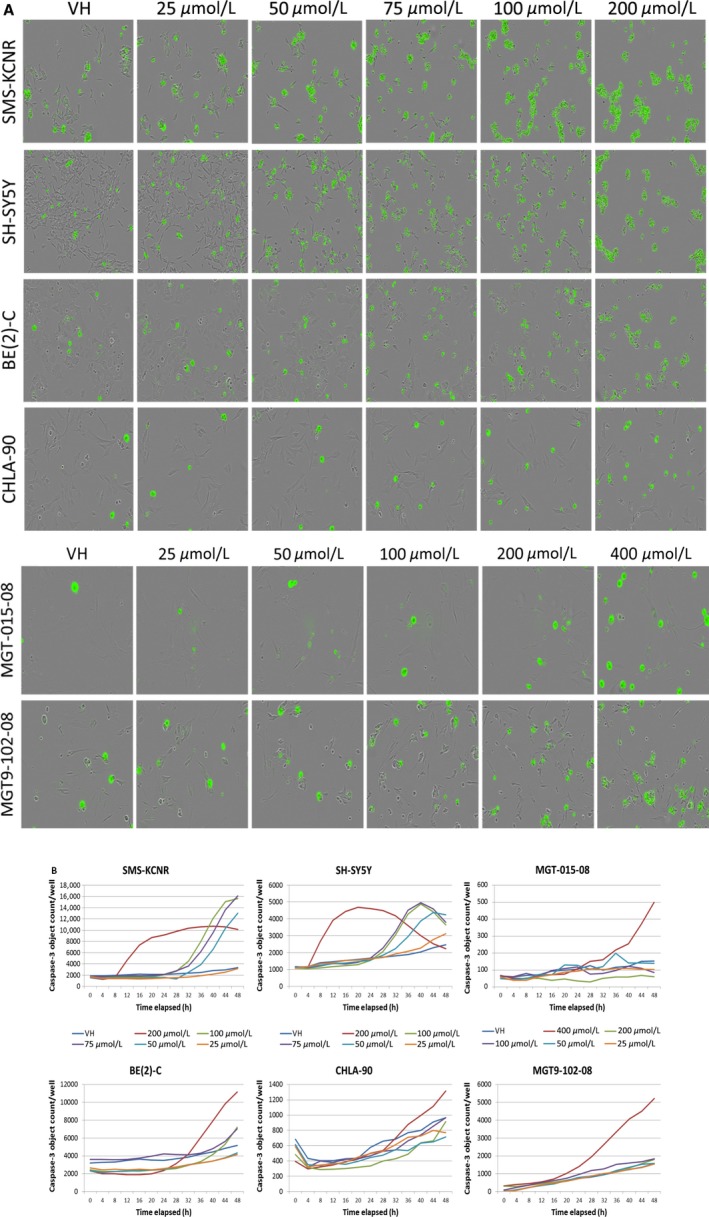
Tolcapone treatment corresponds to a dose‐dependent increase in cell death in NB. (A) Images of established and primary NB cells treated with 200 nmol/L SYTOX and varying concentrations of Tolcapone (0–400 μmol/L) taken with the IncuCyte ZOOM. (B) Quantification of cleaved caspase‐3 expressed in images taken of NB cells treated with 5 μmol/L kinetic caspase‐3 reagent and varying concentrations of Tolcapone (0–400 μmol/L).

Both established and primary NB cell lines were imaged with the IncuCyte ZOOM every 4 h for 48 h using the nucleic acid stain SYTOX Green reagent, which can infiltrate compromised cell membranes associated with dead or dying cells. A dose‐dependent increase in cell death when treated with Tolcapone was seen across all NB cells imaged (Fig. 3A).

### Tolcapone induces caspase‐mediated apoptosis in neuroblastoma

Along with being imaged for cytotoxicity, NB cells were also stained and imaged for caspase‐mediated apoptosis using a kinetic caspase‐3/7 reagent (Essen Bioscience). The number of “caspase‐3 objects” per well was calculated using IncuCyte software and graphed, showing a dose‐dependent increase in the number of cells undergoing caspase‐mediated apoptosis when treated with Tolcapone (Fig. 3B). The trend in caspase‐3/7 activation for different cell lines correlates with their IC_50_ value, with SMS‐KCNR cells being the most vulnerable to Tolcapone and CHLA‐90 being the most resistant established cell line.

**Figure 4:**
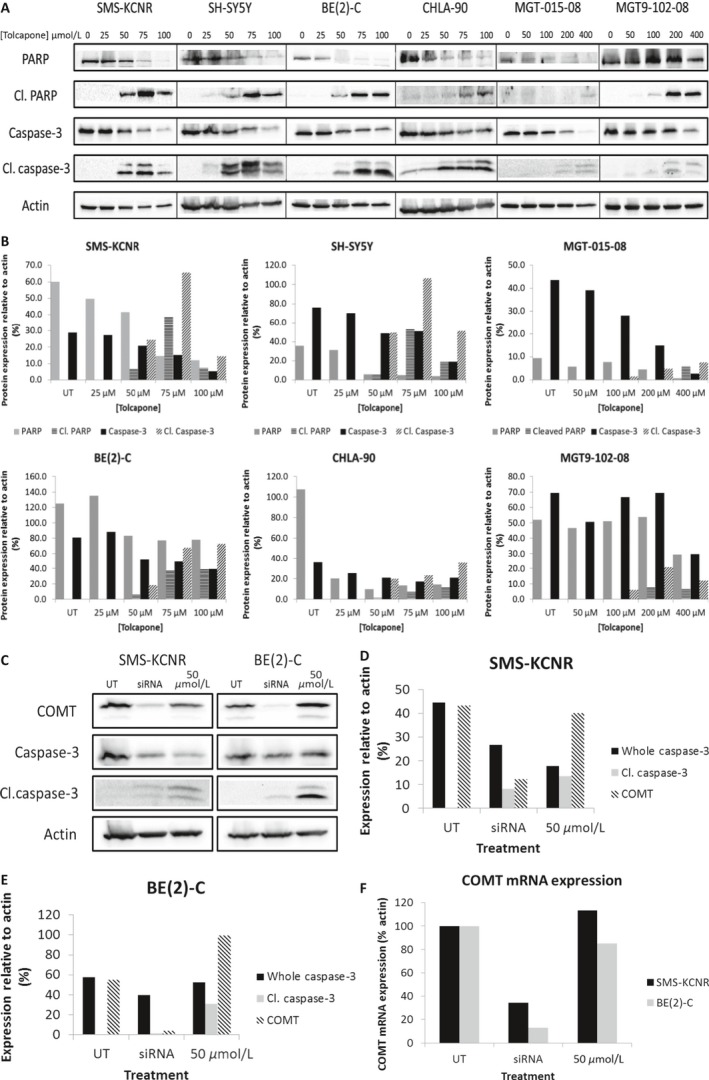
Inhibition of COMT through Tolcapone treatment or siRNA silencing induces apoptosis in NB cell lines. (A) Western blot analyses indicated a dose‐dependent increase in cleaved caspase‐3 and cleaved PARP and a reduction in whole caspase‐3 and whole PARP protein expression as NB cells were introduced to increasing concentrations of Tolcapone drug therapy. (B) Blots were normalized to actin control and quantified as a percent compared to β‐Actin expression. (C) COMT, whole and cleaved caspase‐3 protein expression for both SMS‐KCNR and BE(2)‐C cells treated with siRNA and 50 μmol/L Tolcapone. (D) SMS‐KCNR bands were normalized to β‐Actin control. (E) BE(2)‐C bands were normalized to β‐Actin control. (F) RT‐qPCR analysis confirmed a knockdown of COMT mRNA in SMS‐KCNR and BE(2)‐C cells.

Western Blot analyses further revealed a dose‐dependent increase in cleaved caspase‐3 and cleaved PARP protein in all six NB cell lines and a subsequent decrease in whole caspase‐3 and whole PARP protein, indicating that Tolcapone treatment activates downstream apoptotic events in NB cells (Fig. 4A and B).

### Inhibition of COMT results in apoptosis in Neuroblastoma

To confirm that the inhibition of COMT was mechanistically involved in the death of NB cells treated with Tolcapone, SMS‐KCNR and BE(2)‐C cells were transfected with siRNA targeting COMT. Western blot analyses indicated that the silencing of the COMT gene in NB cells with siRNA leads to a reduction in COMT protein expression, as well as an increased cleaved caspase‐3 expression (Fig. 4C–E). RT‐qPCR confirmed the silencing of COMT in the siRNA‐treated cell line compared to control (Fig. 4F).

### Tolcapone reduces ATP production in NB cells

**Figure 5:**
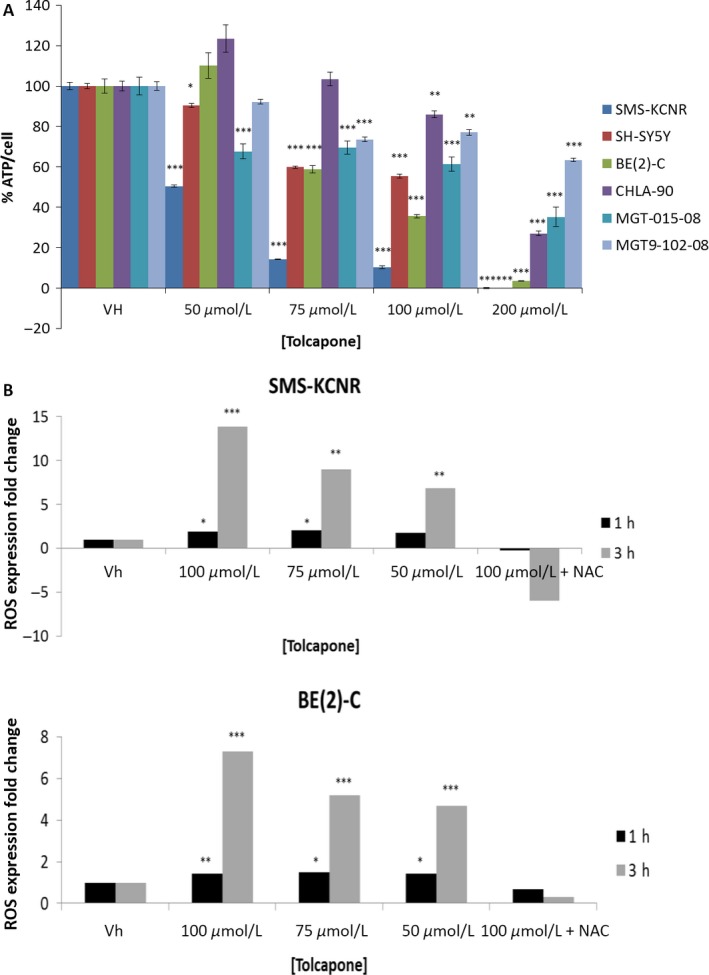
Tolcapone treatment corresponds to a dose‐dependent decrease in cellular metabolic activity and increase in reactive oxygen species (ROS) in NB cell lines. (A) Cells were treated with Tolcapone (0–200 μmol/L) for 48 h. Cell Titer GLO was used to measure total cellular ATP and CyQuant was used to measure total cellular DNA (*n* = 3, graph is representative of one replicate). Values are mean ± SD. (B) Cells were stained with 20 μmol/L DCFDA to measure ROS for 45 min and then treated with varying concentrations of Tolcapone (0–100 μmol/L) with or without NAC for either 1 or 3 h (*n* = 3, graph is representative of one replicate). Values are mean ± SD (*P < 0.05, *^*^ *P* < 0.01, *^**^ *P* < 0.001).

In order to analyze the effect of Tolcapone treatment on metabolic activity in NB cells, ATP levels in treated NB cells were measured by Cell Titer GLO luminescent assay coupled with CyQuant fluorescent DNA assays. All cells lines saw a significant dose‐dependent reduction in ATP when treated with Tolcapone. Tolcapone treatment showed the greatest reduction in ATP‐per‐cell compared to control in SMS‐KCNR and SH‐SY5Y, which is consistent with the cell viability and apoptosis data (Fig. 2A and 5A).

### Tolcapone increases reactive oxygen species in NB

Analysis of reactive oxygen species showed a dose‐dependent increase in ROS for both SMS‐KCNR and BE(2)‐C NB cell lines treated with 0–100 μmol/L Tolcapone for 1 h and 3 h time points (Fig. 5B). A greater ROS fold‐change compared to vehicle control was observed in SMS‐KCNR cells than BE(2)‐C cells, indicative of a greater sensitivity to Tolcapone treatment for SMS‐KCNR cells. This result corresponds to the lower IC_50_ value and greater ATP‐per‐cell reduction observed for SMS‐KCNR cells compared to BE(2)‐C cells. However, the increase in ROS was completely prevented in both SMS‐KCNR and BE(2)‐C cells that were treated with N‐acetylcysteine (2.5 mmol/L) prior to being exposed to 100 μmol/L Tolcapone.

### Tolcapone inhibits tumor growth and prolongs survival in vivo

**Figure 6:**
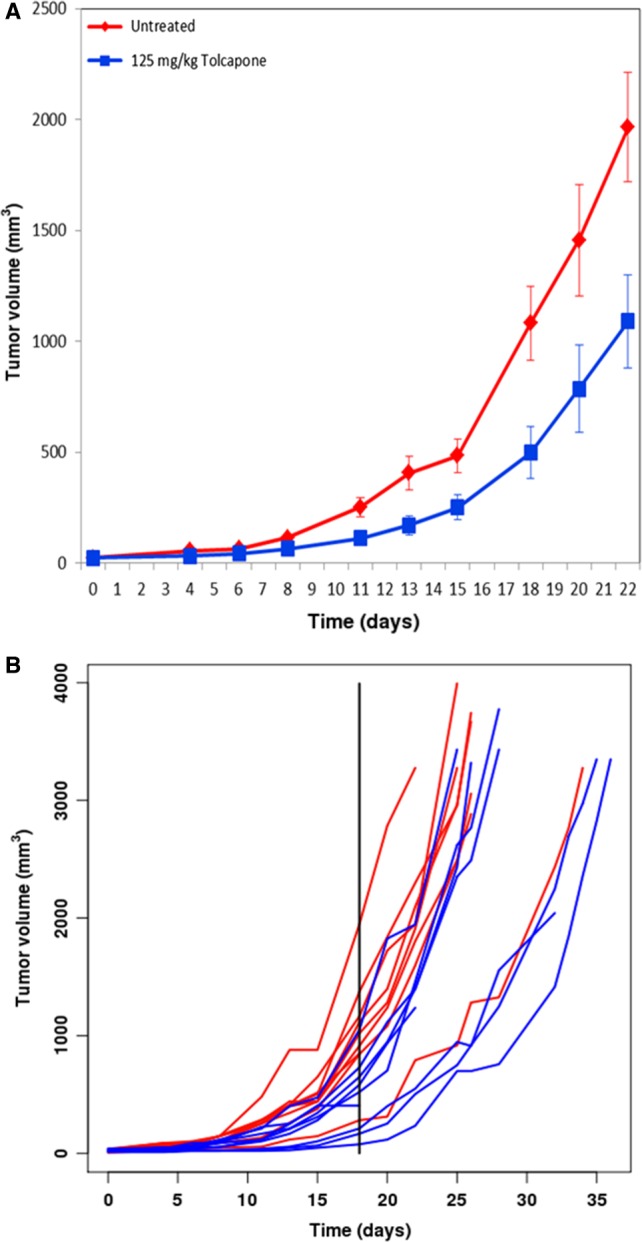
Treatment with Tolcapone significantly reduced tumor volume compared to control in vivo. Mice were injected with 2 million SMS‐KCNR NB cells and treated orally with 125 mg/kg Tolcapone every 24 h. A) Mean tumor volume of both treatment groups over time (one replicate shown, *P* < 0.05). B) Growth pattern of mouse tumors over time with each line representing a specific mouse in the experiment (one replicate shown, *P* < 0.05).

In vivo studies using SMS‐KCNR xenograft models demonstrated that Tolcapone as a single agent therapy decreases tumor volume compared to control (*P* < 0.01, based on three replicate experiments with nine mice per group in each replicate). In one such experiment (Fig. 6A), Tolcapone treatment of 125 mg/kg resulted in a smaller average tumor of 490 ± 310 mm^3^ compared to control tumors of 1100 ± 450 mm^3^ (Fig. 6B). There were no adverse events or differences in weight or behavior noted in the mice.

## Discussion

For the first time, this study investigates the mechanistic and therapeutic effects of Tolcapone treatment in multiple neuroblastoma cell lines and patient‐derived tumor cells. The genomic sequencing of tumor biopsies from patients with NB showing upregulation of COMT uncovered this potentially novel therapy not previously considered in the past. Tolcapone induced apoptotic cell death in all NB cell lines. Furthermore, a reduction in ATP level per cell and increase in ROS when NB cells are treated with Tolcapone provides support for the hypothesized mechanism, in which COMT inhibition leads to an overexpression of dopamine, a subsequent release of ROS, and programmed cell death in NB. In vivo tumor reduction and prolonged survival for mice treated with Tolcapone compared to control provide additional support for the potential use of Tolcapone for the treatment of patients with neuroblastoma.

The data acquired with the IncuCyte ZOOM indicate that the apoptotic response of NB cells increases in a dose‐dependent manner with Tolcapone treatment (0–200 μmol/L; Fig. 3). SMS‐KCNR and SH‐SY5Y showed a sharp increase in caspase‐3 activation in the initial hours of Tolcapone treatment, which then plateaued and declined in the later hours of treatment (Fig. 3). This is likely due to the high sensitivity of these cell lines to Tolcapone relative to the other NB cells tested as evidenced by their lower IC_50_ values (Fig. 2).

An increase in apoptotic markers was seen in SMS‐KCNR and BE(2)‐C cells when COMT was silenced with siRNA, suggesting that the inhibition of COMT—via Tolcapone treatment or silencing of the COMT enzyme—is mechanistically involved in the induction of apoptosis in NB cells. There was a greater increase in cleaved caspase‐3 expression in SMS‐KCNR cells compared to BE(2)‐C (Fig. 4D and G). This observation, along with our results from treating these same cells with Tolcapone, indicates that SMS‐KCNR cells are more sensitive to the inhibition of COMT whether it is achieved through Tolcapone treatment or siRNA treatment. However, it is important to note that one study found that Entacapone, another COMT inhibitor, did not induce cell death in SH‐SY5Y NB cells 20. This could be due to the suggestion that Entacapone is not as potent of an inhibitor as Tolcapone or that Tolcapone could be inducing apoptosis through COMT inhibition as well as another mechanism 21, 22. Notably, Entacapone has not been shown to cause alterations in liver function or liver damage as have been observed in patients that received Tolcapone treatment 22.

To further elucidate the downstream mechanism by which Tolcapone inhibits NB proliferation and induces apoptosis, this study sought to analyze the production of ROS in response to Tolcapone treatment. Our findings indicate a dose‐dependent increase in ROS with Tolcapone treatment in both BE(2)‐C and SMS‐KCNR cells. This result is consistent with those previously reported by Ouazia et al. (2015), which showed an increase of ROS when NB cells were treated with DA 9. The dose‐dependent increase in ROS positively correlates with cell death among NB cells when treated with Tolcapone, providing support for ROS production as one mechanism by which Tolcapone is toxic to NB cells. Previous studies have also seen that sharp increases in ROS induce apoptosis in cancers other than NB 23, 24, 25, 26.

The data in this paper support the Korlipara et al. (2004) findings that Tolcapone treatment inhibits cell proliferation and reduces ATP in cells. Both established cell lines and preliminary cell lines showed a cytotoxic response to Tolcapone treatment in vitro, where tumors varied in sensitivity to drug treatment (Fig. 2A). Furthermore, ATP per cell production showed a dose‐dependent decrease at increasing doses of Tolcapone across all NB tumor cell lines. According to the widely studied “Warburg Effect”, many cancer cells—including NB cells—are reprogrammed to produce ATP via glycolytic metabolism 27, 28, 29, 30. Therefore, decreased ATP‐per‐cell activity in NB cells after Tolcapone treatment suggests that the single‐agent therapy may work as a glycolytic inhibitor (Fig. 5B).

Interestingly, two previous studies reported that the silencing of COMT in colorectal and pancreatic cancer resulted in a decrease in apoptosis 31, 32. These conflicting results suggest that NB responds differently to COMT inhibition compared to colorectal or pancreatic cancer, which may be attributed to the high expression of DA—eliciting the release of ROS when highly overexpressed—in NB compared to other malignancies 4, 5. With seemingly differing effects in multiple malignancies, targeting COMT's role may be an important strategy to provide treatment or understand mechanistic changes in tumors.

Consistent with the in vitro findings, in vivo analysis provided support for the potential efficacy of Tolcapone treatment in pediatric patients with NB. This was indicated by a significant reduction in tumor size for mice treated with Tolcapone compared to control repeated in triplicate. While dopamine changes may affect motivation and attention, the mice treated with Tolcapone exhibited no noticeable changes in behavior or showed any additional negative side effects compared to the control mice.

Taken together, these data support the possibility of Tolcapone being a promising novel therapy for the treatment of neuroblastoma. The preclinical data presented in this paper provided the support needed to launch a phase I clinical trial treating neuroblastoma patients with Tolcapone within the Neuroblastoma and Medulloblastoma Translational Research Consortium. The trial is the first of its kind, using Tolcapone both in a pediatric setting and as a treatment for cancer.

## Conflict of Interest

None declared.
